# Crystal Structure of a Two-Subunit TrkA Octameric Gating Ring Assembly

**DOI:** 10.1371/journal.pone.0122512

**Published:** 2015-03-31

**Authors:** Marc C. Deller, Hope A. Johnson, Mitchell D. Miller, Glen Spraggon, Marc-André Elsliger, Ian A. Wilson, Scott A. Lesley

**Affiliations:** 1 The Joint Center for Structural Genomics, and Department of Integrative Structural and Computational Biology, The Scripps Research Institute, La Jolla, California, United States of America; 2 The Joint Center for Structural Genomics, and Stanford Synchrotron Radiation Lightsource, SLAC National Accelerator Laboratory, Menlo Park, California, United States of America; 3 Protein Sciences Department, Genomics Institute of the Novartis Research Foundation, San Diego, California, United States of America; Zhejiang University, CHINA

## Abstract

The *TM1088* locus of *T*. *maritima* codes for two proteins designated TM1088A and TM1088B, which combine to form the cytosolic portion of a putative Trk K^+^ transporter. We report the crystal structure of this assembly to a resolution of 3.45 Å. The high resolution crystal structures of the components of the assembly, TM1088A and TM1088B, were also determined independently to 1.50 Å and 1.55 Å, respectively. The TM1088 proteins are structurally homologous to each other and to other K^+^ transporter proteins, such as TrkA. These proteins form a cytosolic gating ring assembly that controls the flow of K^+^ ions across the membrane. TM1088 represents the first structure of a two-subunit Trk assembly. Despite the atypical genetics and chain organization of the TM1088 assembly, it shares significant structural homology and an overall quaternary organization with other single-subunit K^+^ gating ring assemblies. This structure provides the first structural insights into what may be an evolutionary ancestor of more modern single-subunit K^+^ gating ring assemblies.

## Introduction

K^+^ ions are the major monovalent cation in prokaryotic and eukaryotic cells and stringent regulation of its intracellular concentration is essential for a wide variety of cellular processes [1]. Ion transporters that translocate cations, particularly K^+^, are central to the regulation of cellular pH and osmolarity [2], electrical signal generation [3] and regulation of protein expression and activity [4].

In contrast to the three K^+^ transporters found in *E*. *coli* (Kdp, Kup and Trk), only one K^+^ transport system, Trk, has been identified in *T*. *maritima* [5]. Trk is a member of the Trk/Ktr/HKT family of transporters, which are specific to non-animal cells [6]. The Trk system is the major constitutive K^+^ transporter in *E*. *coli* and functions as a proton symporter powered by the proton motive force and binding of ATP [7,8]. The *E*. *coli* Trk potassium transporter complex consists of a cytosolic regulatory component, TrkA, which associates with the channel forming membrane component, TrkH. In *T*. *maritima* the cytosolic regulatory component of Trk comprises of two proteins, TM1088A and TM1088B, that associate with the TrkH component, TM1089, to form the functional transporter [9].

Given the requirement for tight regulation of K^+^, several common structural features have evolved, including the use of conserved cytoplasmic regulatory components for control of ion flow through the transmembrane channel. One such feature is the RCK domain (Regulating the Conductance of K^+^) that is present in a large class of proteins involved in the regulation of many eukaryotic and prokaryotic K^+^ transporters [10]. More than half of the prokaryotic K^+^ channels identified so far are associated with RCK domains, making them the most common regulatory domain of prokaryotic K^+^ channels [11,12]. A sub-family of RCK regulatory proteins, including those found in prokaryotic KefC, KtrA and TrkA channels, have homology to dinucleotide binding proteins. These RCK family members are often collectively referred to as KTN (K^+^ Transport, Nucleotide-binding) domains due to their ability to bind nucleotides. KTN and RCK domains are closely related and both adopt a Rossmann-like fold (β-α-β-α-β) [11,13,14].

Several structural studies have shown that RCK domains form homodimeric assemblies arranged in a clamshell-like arrangement with a hinge region formed by two domain-swapped helices. This arrangement is observed in many crystal structures including the *M*. *thermoautotrophicum* potassium channel (MthK)[15–18], *M*. *jannaschii* TrkA [19], *B*. *subtilis* KtrA [19,20], *E*. *coli* K^+^ channel [10], *E*. *coli* ybaL, *V*. *parahaemolyticus* TrkA [21] and *Danio rerio* Ca^2+^-activated K^+^ channel (BK)[22]. Structural and biochemical studies have also shown that these RCK homodimers assemble into higher-order quaternary structures to form cytosolic regulatory components referred to as gating ring assemblies, which are typically composed of a tetrameric arrangement of protein chains, with each chain itself composed of an RCK dimer. This assembly typically results in a symmetrical octameric arrangement of RCK domains that form a central pore on the cytosolic side of the channel.

Several crystal structures of K^+^ transporter gating ring assemblies have been determined including MthK [16,18], KtrA [20,23], BK [22,24], GsuK [25] and TrkA [21]. The gating ring assembly combines with the membrane spanning units to form a functional transporter complex. The RCK domains of the gating ring assembly undergo conformational rearrangements in their dimeric associations in response to external stimuli, such as Ca^2+^ and nucleotides. These conformational changes are symmetrical in nature and involve an expansion or contraction of the central ring of RCK domains, which propagate to the associated membrane-spanning channel resulting in regulation of ion flow across the membrane [21,22]. For example, the BK channel gating ring assembly has been eloquently described as undergoing a conformation change that opens the channel “like the petals of a flower” [22].

Previous protein expression experiments and phylogentic analysis conducted in our lab have shown that the *TM1088* locus of *T*. *maritima* codes for two proteins designated as TM1088A and TM1088B, which combine to form an octameric assembly [9]. The octameric TM1088 structure is the first example of a two-subunit Trk K^+^ transporter composed of two different proteins. This two-subunit complex is produced from a single promoter, as a result of overlapping start and stop codons, and an internal ribosome binding site within the *TM1088* locus. This scenario is suggestive of a gene duplication event similar to that proposed in the evolution of *E*. *coli TrkA* and other Trk/KtrAB proteins [14,26].

Given the somewhat atypical genetics and protein composition of the TM1088 assembly, it is remarkable to find that the structure conforms to the structural paradigms observed in all other RCK-containing K^+^ transporters studied to date. Similarities of the TM1088 assembly to other gating ring structures are highlighted, along with some unique features including an interfacial salt bridge across the central cavity and several protein interfaces with implications for gating ring assembly and mechanism. This structure provides a framework for future structural and functional studies on the mechanisms of K^+^ ion regulation by this two-subunit family of K^+^ transporters.

## Materials and Methods

### Strains, plasmids, recombinant techniques, expression and affinity purification

**Fig 1:**
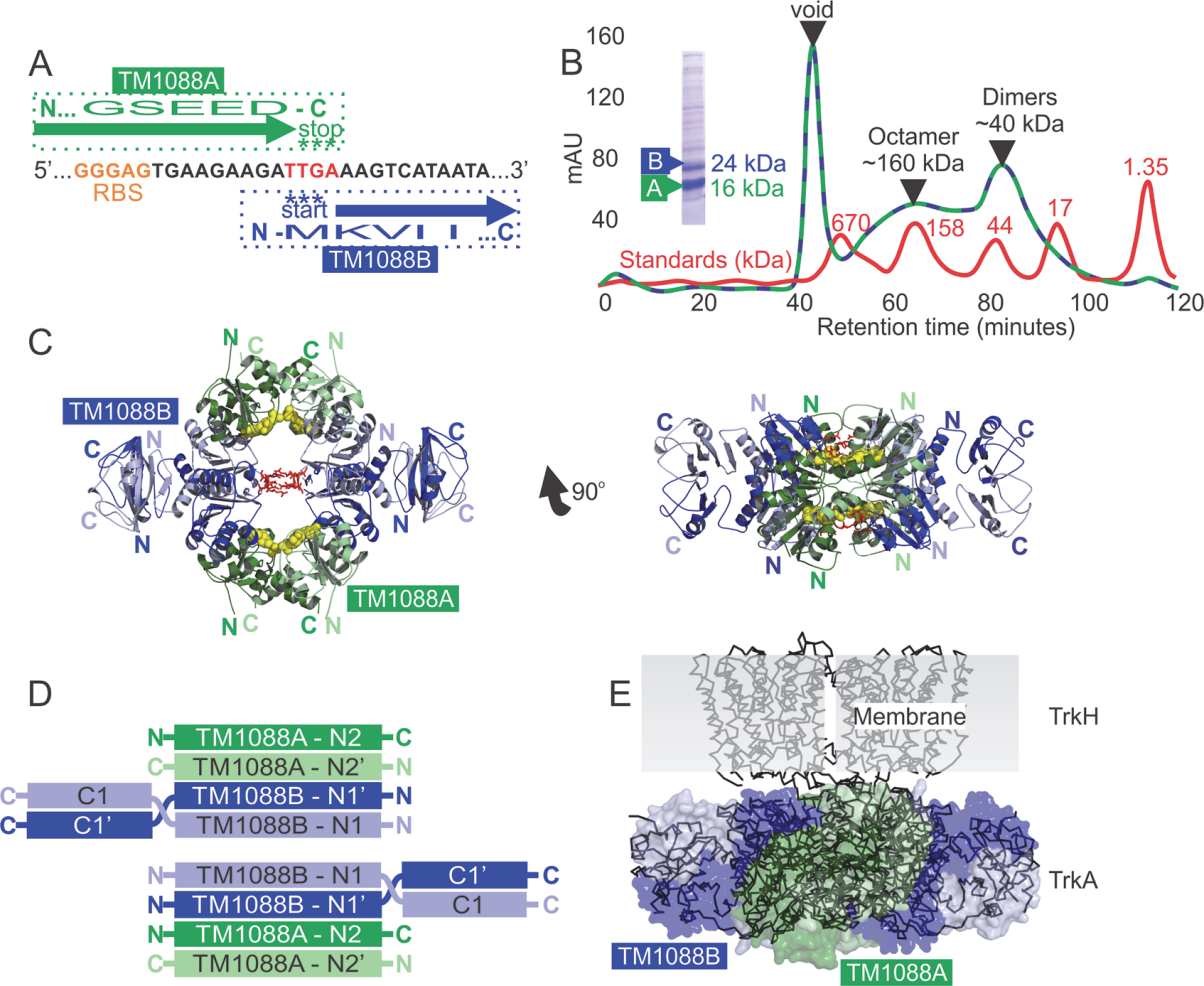
The *TM1088* locus codes for two proteins that form an octameric assembly. (A). *TM1088* locus in the region of the overlapping start (TTG) and stop (TGA) codons (shown in red) of TM1088B and TM1088A, respectively (GenBank: NP_228894). The putative Ribosome Binding Site (RBS; GGGAG) is shown in orange. The C-terminal portion of the TM1088A transcript is shown in green (UniProt: D3KFX7_THEMA) and the N-terminal portion of the TM1088B transcript is shown in blue (UniProt: D3KFX8_THEMA). (B) Purification of the octameric TM1088 complex using size exclusion chromatography. Molecular weight standards (shown in red) and TM1088 sample (shown in dashed green-blue) were run on an S200 column at 0.5 ml/min. SDS-PAGE of affinity purified sample prior to final size exclusion step is shown inset. (C) Ribbon representation of the octameric TM1088 assembly. TM1088A and TM1088B are shown in green and blue, respectively. Bound AMP is shown as yellow spheres and residues involved in hydrogen bonding across the central cavity are highlighted by red sticks (Arg33, Glu34 and Glu37). (D) Schematic representation of the domain organization of the octameric TM1088 assembly. (E) Superposition of the octameric TM1088 assembly on to TrkA/TrkH of *V*. *parahaemolyticus* (PDB ID: 4J9U) demonstrating the overall similarity in size and architecture between the two structures. The transparent molecular surface of TM1088A and TM1088B is shown in green and blue, respectively, and the Cα ribbon trace of TrkA/TrKH in black.

The *TM1088* locus (GenBank: NP_228894) consists of two separate open reading frames with overlapping start and stop codons and, as such, encodes two separate proteins designated TM1088A (UniProt: D3KFX7_THEMA, Pfam02254) and TM1088B (UniProt: D3KFX8_THEMA, Pfam02254 and Pfam02080)(Fig 1A). In some databases TM1088A is, however, annotated as locus TM_1087.1 and TM1088B as locus TM_1088. For the purpose of this study, plasmid pMH4-TM1088 was used to produce TM1088A:TM1088B complexes (TM1088A: full length, residues 1–143 and TM1088B: full length, residues 1–218) and TM1088A (full length, residues 1–143) through use of different purification schemes. Plasmid pMH4-TM1088 was constructed as previously described as part of our structural genomics exploration into the entire *T*. *maritima* proteome [27]. Plasmid pSpeedET-TM1088B was used to produce TM1088B (residues 140–218) as previously described [9].

pMH4-TM1088 was constructed by amplification of the *TM1088* locus using Polymerase Chain Reaction (PCR) from *T*. *maritima* MSB8 (DSM3109) genomic DNA using *PfuTurbo* DNA polymerase (Stratagene) and primers corresponding to the predicted 5' (5’-CTGTACTTCCAGGGCATGTCAAAAAAACAAAAAAGC-3’) and 3' (5’-AATTAAGTCGCGTTATCAACTATTCACACCTCC-3’) ends. The PCR product was cloned into plasmid pMH4, which encodes an expression and purification tag (MGSDKIHHHHHH) at the N-terminus of the full-length TM1088A protein. The expression and purification tag was not cleaved from the final product. The cloning junctions were confirmed by DNA sequencing. Expression was performed in a modified Terrific Broth (TB) using the *E*. *coli* strain GeneHogs (Invitrogen). At the end of fermentation, lysozyme was added to the culture to a final concentration of 250 μg/ml, and the cells were harvested and frozen. After one freeze/thaw cycle, the cells were sonicated in lysis buffer (50 mM Tris pH 7.9, 50 mM NaCl, 10 mM imidazole, 1 mM Tris(2-carboxyethyl)phosphine-HCl (TCEP)) and the lysate was clarified by centrifugation at 32,500 x g for 30 minutes. The soluble fraction was passed over nickel-chelating resin (GE Healthcare) pre-equilibrated with lysis buffer, the resin washed with wash buffer (50 mM Tris pH 7.9, 300 mM NaCl, 40 mM imidazole, 10% (v/v) glycerol, 1 mM TCEP), and the protein was eluted with elution buffer (20 mM Tris pH 7.9, 300 mM imidazole, 10% (v/v) glycerol, 1 mM TCEP). The eluate was diluted 10-fold with buffer Q (20 mM Tris pH 7.9, 5% (v/v) glycerol, 0.25 mM TCEP) containing 50 mM NaCl and loaded onto a RESOURCE Q column (GE Healthcare) pre-equilibrated with the same buffer. Both TM1088A and the TM1088 complex were eluted with a linear gradient of 50–500 mM NaCl in buffer Q, buffer exchanged with crystallization buffer (20 mM Tris pH 7.9, 150 mM NaCl, 0.25 mM TCEP), and further purified on a 1.6 x 60 cm^2^ HiLoad Superdex 200 column (GE Heathcare) with isocratic elution in Tris crystallization buffer (20 mM Tris pH 7.9, 150 mM NaCl, 0.25 mM TCEP). TM1088A and the TM1088 complex fractions were concentrated and subjected to crystallization trials.

pSpeedET-TM1088B was generated using the Polymerase Incomplete Primer Extension (PIPE) cloning method (Klock et al., 2008). The *TM1088B* C-terminal domain (residues 140–218) was amplified by PCR from *T*. *maritima* MSB8 (DSM3109) genomic DNA using *PfuTurbo* DNA polymerase (Stratagene) and I-PIPE (Insert) primers (forward primer, 5’-ctgtacttccagggcATGAAAGTCATAATAATCGGTGG-3’ and reverse primer, 5’-aattaagtcgcgttaTCAGAACTCATCTGGGAAGATGAGAGC-3’, target sequence in upper case) that included sequences for the predicted 5' and 3' ends. The expression vector, pSpeedET, which encodes an amino-terminal tobacco etch virus (TEV) protease-cleavable expression and purification tag (MGSDKIHHHHHHENLYFQ/G), was PCR amplified with V-PIPE (Vector) primers (forward primer: 5’-taacgcgacttaattaactcgtttaaacggtctccagc-3’, reverse primer: 5’-gccctggaagtacaggttttcgtgatgatgatgatgatg-3’). V-PIPE and I-PIPE PCR products were mixed to anneal the amplified DNA fragments together. *E*. *coli* GeneHogs (Invitrogen) competent cells were transformed with the I-PIPE / V-PIPE mixture and dispensed on selective LB-agar plates. The cloning junctions were confirmed by DNA sequencing. Expression was performed in *E*. *coli* HK100 cells grown at 37^°^C in TB medium containing kanamycin. Cultures were grown to OD_600_ = 0.5 then induced with 0.02% (w/v) arabinose. After 4 hours, the cultures were harvested by centrifugation at 2,000 x g for 20 minutes. Cell pellets were resuspended in 20 mM TRIS pH 8, 0.1M NaCl, 0.5 mM TCEP and disrupted by a microfluidizer (Microfluidics). Unbroken cells and cellular debris were removed by centrifugation at 12,000 x g for 20 minutes. The supernatant was applied to a 1 mL Ni-NTA agarose (Qiagen) gravity column equilibrated with 50 mM TRIS pH 8, 0.05 M NaCl, 10 mM imidazole, 0.5 mM TCEP. The column was washed with 50 mM TRIS pH 8, 0.3 M NaCl, 40 mM imidazole, 10% glycerol, 0.5 mM TCEP. The His-tagged TM1088B C-terminal protein was then eluted with 20 mM TRIS pH 8, 0.3 M imidazole, 10% glycerol, 0.5 mM TCEP, concentrated and then submitted for crystallization trials with the tag intact.

Protein concentrations were determined by the Bradford method using a commercially available assay (Pierce) with bovine serum albumin as standard. SDS-PAGE was performed as described [28].

### Crystallization and data collection

TM1088A was concentrated to 10 mg/ml using centrifugal ultrafiltration (Millipore). TM1088A was crystallized using the nanodroplet vapor diffusion method with standard JCSG crystallization protocols [27,29]. Sitting drops composed of 200 nl protein solution mixed with 200 nl crystallization solution in a sitting drop format were equilibrated against a 50 μl reservoir at 293 K for 54 days prior to harvest. The crystallization reagent consisted of 40% MPD, 5% PEG-8000, and 0.1M Cacodylate pH 6.5. No further cryoprotectant was added to the crystal. Initial screening for diffraction was carried out using the Stanford Automated Mounting system (SAM) at the Stanford Synchrotron Radiation Lightsource (SSRL, Menlo Park, CA).

The TM1088B C-terminal domain was dialyzed into crystallization buffer (20 mM Tris, pH 7.8 and 150 mM NaCl) and concentrated to 15 mg/ml using centrifugal ultrafiltration (Millipore). TM1088B C-terminal domain was crystallized using the nanodroplet vapor diffusion method with standard JCSG crystallization protocols [27,29]. Sitting drops composed of 150 nl protein solution mixed with 150 nl crystallization solution in a sitting drop format were equilibrated against a 60 μl reservoir at 293 K. The crystallization reagent consisted of 25% (v/v) ethylene glycol. No further cryoprotectant was added to the crystal.

The TM1088 complex was concentrated to 3 mg/ml using centrifugal ultrafiltration (Millipore) and NADH added to a final molar ratio of 1.1:1.0 (NADH: protein). The TM1088 complex was crystallized by vapor diffusion against 0.1 M magnesium chloride, 15% (w/v) PEG 4000, 0.1 M HEPES, pH 7.0. Drops consisted of 0.5 μl protein, 0.5 μl reservoir and 0.25 μl xylitol (30% (w/v)) as cryoprotectant with a 60 μl reservoir volume. Reservoirs were overlaid with 10 μl of silicon oil and paraffin oil (1:1) to regulate the rate of vapor diffusion [30].

Crystals were flash-cooled to 100K and X-ray data collected at the Advanced Light Source (ALS, Lawrence Berkeley National Laboratory) at beamlines 5.0.1 (TM1088A) or 5.0.3 (TM1088B and TM1088 complex). All data were processed with HKL2000 or Mosflm and SCALA from the CCP4 package [31,32].

### Phasing, model building and refinement

Refinement was performed with Refmac5 [33] and PHENIX [34–36] and model building with O [37], Coot [38] and MIFit (http://code.google.com/p/mifit). All other crystallographic manipulations were carried out with the CCP4 package [32]. Molecular graphics were prepared using PyMOL (Schrödinger, LLC). Data collection and refinement statistics are shown in Table 1.

**Table 1:** Crystallographic data collection and refinement.

	TM1088 assembly	TM1088A	TM1088B C-terminal domain
PDB ID	3L4B	2G1U	3JXO
Beamline	ALS 5.0.3	ALS 5.0.1	ALS 5.0.3
Space group	P3_2_21	P2	C222_1_
Cell dimensions (Å)	a = b = 96.78, c = 305.10	a = 47.86, b = 35.14, c = 56.58,β = 111.85°	a = 62.11, b = 64.40, c = 97.00
Wavelength (Å)	1.000	1.000	1.000
Resolution range (Å)	48.4–3.45	44.4–1.50	50.0–1.55
Highest resolution shell (Å)	3.57–3.45	1.54–1.50	1.61–1.55
R_merge_ ^+^ (%)^a^	17.9 (77.2)	4.8 (34.4)	6.5 (51.9)
Mean I/σ(I)^a^	11.4 (2.0)	19.1 (1.6)	39.9 (2.3)
Observations	178,674	133,164	395,401
Unique reflections^a^	22,866 (2244)	26,312 (1298)	28,472 (2651)
Reflections used in refinement (R_cryst_)	20,333	24,978	25,563
Reflections used for R_free_	1164	1332	1438
Completeness (%)^a^	99.9 (100.0)	93.4 (63.8)	99.3 (94.1)
R_cryst_ † (R_free_) *(%)	25.9 (30.8)	15.3 (17.5)	15.8 (18.7)
No. protein atoms	8952	1144	2433
No. water molecules	0	110	89
No. hetero atoms	93	40	0
RMSD bond lengths (Å)	0.006	0.016	0.014
RMSD bond angles (^o^)	0.97	1.65	1.48
Ramachandran favored^b^ (outliers)(%)	95.1 (0.9)	99.3 (0)	98.8 (0)
Mean overall protein B-value (AMP)	49.3 (78.7)	18.2 (22.1)	23.2

**+** R_merge_ = Σ_hkl_Σ_i_ | I_i_(hkl)—< I(hkl)> | / Σ_hkl_Σ_i_ I_i_(hkl)

**†** R_cryst_ = Σ||Fobs|-|Fcalc|| / Σ|Fobs| where F_calc_ and F_obs_ are the calculated and observed structure factor amplitudes, respectively.

* R_free_ = as for R_cryst_, but for 5.0% of the total reflections chosen at random and omitted from refinement.

^a ^Numbers in parentheses are for highest resolution shell.

^b^ as determined by MolProbity [39].

Data from two isomorphous crystals of the TM1088 complex were merged together for structure determination by MR as implemented by Phaser [40,41], using the high resolution structure of TM1088A (PDB ID: 2G1U) as a search model. During the search for the first monomer, the two possible enantiomorphic space groups were tested, revealing a clear peak for P3_2_21. Searches for the remaining 7 molecules were conducted in this space group. Refinement included 1 TLS group for each of the 12 distinct protein domains observed in the asymmetric unit (4x TM1088A domains and 8x TM1088B domains) and the same domain definitions were used for application of tight NCS restraints. The TM1088 complex was refined to a resolution of 3.45 Å and converged at R_cryst_ of 25.9% and R_free_ of 30.8%. The difference between R_cryst_ and R_free_ of 4.9% is in excellent agreement with an expected value of 4.97% calculated for this resolution according to Urzhumtsev et al. (*μ*(ΔR) ~ 0.024 ln *d*_PDB_ + 0.020)[42]. Additionally, analysis using the script “phenix.r_factor_statistics” in the package PHENIX [34–36] reports that R_cryst_ and R_free_ values both fall on the peak of the distribution observed for all PDB structures in this resolution range (3.35–3.55 Å). The final model contains 4 TM1088A molecules (Ser7 to Ile138), 4 TM1088B molecules (Met1 to Leu216), 4 AMP molecules and 1 unknown ion modeled as UNX in the asymmetric unit (ASU). It is important to note that all eight protein chains of the octameric TM1088 complex are observed within the ASU, and as such, it is likely that the complex is reflective of a true octameric assembly rather than an artifact of crystal packing. The model has good geometry throughout with the Ramachandran plot produced by MOLPROBITY [39] showing 95.1% of the main chain torsion angles in the favored regions with 0.9% outliers. These outliers comprise TM1088B residues Gly8, Ile139 and Leu142, which, with the exception of Leu142, lie in regions not covered by the high-resolution structure of TM1088B, and therefore contribute to more difficulty in the model building and refinement of these residues at this resolution.

The TM1088A structure was determined by MR (search model PDB ID: 1LSS) in space group P2 using MOLREP [43] and the JCSG MR pipeline [44] and refined at 1.5 Å resolution using Refmac [33]. Refinement included 4 TLS groups as determined by the TLS Motion Determination server [45]. The refinement converged at an R_cryst_ of 15.4% and R_free_ of 17.5%. The model consists of Lys4 to Ser140, 1 AMP, 2 MPD, 1 sodium ion and 110 water molecules in the ASU. Met(-11) to Lys3 and Glu141 to Asp143, as well as side-chain atoms of Lys4, Glu49, Lys66, Glu100, Lys113, Lys134 and Ser140 were omitted due to weak or absent electron density. The model has excellent geometry throughout with the Ramachandran plot produced by MOLPROBITY [39] showing 99.3% of the main-chain torsion angles in the favored regions with no outliers.

The structure of the C-terminal domain of TM1088B was determined by MR (search model, PDB ID: 2G1U) in space group C222_1_ and refined to 1.55 Å using Refmac [33]. Refinement included 1 TLS group for each protein chain in the ASU. The refinement of the structure converged at an R_cryst_ of 15.8% and R_free_ of 18.7%. The model consists of Ile140 to Leu216 in chain A, Ile140 to Gly217 in chain B, and 89 water molecules in the ASU. Both protein molecules in the ASU contain additional residues, Asn(-7) to Met(-1), from the uncleaved expression and purification tag. Side-chain atoms from Asn133, Lys162 and Lys208 of chain A and Asn133, Gln144, Glu155, Asp156, Arg171, Glu206 and Glu209 of chain B were omitted due to weak or absent electron density. The model has excellent geometry throughout with the Ramachandran plot produced by MOLPROBITY [39] showing 98.8% of the main-chain torsion angles in favored regions with no outliers.

## Results and Discussion

### The *TM1088* locus codes for two proteins that form an octameric assembly

TM1088A and TM1088B were previously identified and functionally characterized as a two-subunit Trk K^+^ transporter of *T*. *maritima* [9]. Expression of the *TM1088* locus resulted in two distinct, but structurally homologous, proteins designated TM1088A (16.0 kDa, Pfam ID: PF02254) and TM1088B (24.0 kDa, Pfam ID: PF02254 and Pfam ID: PF02080, Fig 1A, n.b. TM1088A is colored green and TM1088B blue in all figures). TM1088A and TM1088B were purified and crystallized as an octameric assembly consisting of 4 units of TM1088A and 4 units of TM1088B (160 kDa octameric complex; Fig 1B–1C)[9]. The differential expression of TM1088A over TM1088B (~2-fold) was previously reported as shown here in Fig 1B (SDS-PAGE gel, inset)[9]. The lower yield observed for TM1088B may be a result of lower expression from the internal RBS or dissociation of the untagged TM1088B from the tagged TM1088A during affinity purification. The latter scenario is supported by SEC analysis, which confirms that the octameric complex is in equilibrium with lower stoichiometries of the complex including dimers (Fig 1B).

A schematic representation of the domain structure of the octameric TM1088 complex is shown in Fig 1D. TM1088A is composed of a single N-terminal Trk domain that is annotated as Pfam ID: PF02254 (Fig 1D, N2 and N2’, where the “prime” nomenclature is used to denote partner pairs within a dimer). Similarly, TM1088B is also composed of an N-terminal Trk domain, that is also annotated as Pfam ID: PF02254 (Fig 1D, N1 and N1’) and a second smaller C-terminal Trk domain annotated as Pfam ID: PF02080 (Fig 1D, C1 and C1’).

The crystal structure of the TM1088 octameric assembly was determined to 3.45 Å using molecular replacement (Fig 1C and Table 1, PDB ID: 3L4B). This resolution is similar to other K^+^ gating ring assemblies which have been reported to 2.7 Å and 3.5 Å (KtrAB, PDB ID: 2HMS and 4J7C)[20], 2.79 Å (MthK, PDB ID: 2FY8)[16,18], 3.61 Å and 3.1 Å (BK, PDB ID: 3U6N and 3NAF)[22,24], 3.7 Å (GsuK, PDB ID: 4GX5)[25] and 3.80 Å and 3.05 Å (KtrA/TrkH, PDB ID: 4J9U and 4J9V)[21]. The octameric TM1088 assembly has significant sequence and structural homology with the single-subunit TrkA gating ring assembly of *V*. *parahaemolyticus* (PDB ID: 4J9U)[21]. Specifically, TM1088A and TM1088B share 29% and 22% sequence identity with TrkA, respectively, as well as considerable structural homology for the individual domains. However, all atom superpositions of the octameric TM1088 assembly with the TrkA assembly results in an RMSD of 9.0 Å over 5136 atoms (Fig 1E) due to differences in domain orientations between the two structures. The overall similarity in quaternary structure would position TM1088 adjacent to the cell membrane in a position suitable for association with its partner protein TM1089 (Fig 1E, TrkH is homologous to TM1089). Using *in vivo* complementation assays, both *TM1088* and *TM1089* have been shown to be required for cell survival in low potassium [9]. The high level of structural homology with the TrkA assembly is consistent with TM1088 being a member of the Trk family of transporters, which in turn, is consistent with the annotations in all the major protein family databases including Pfam [46].

### Crystal structures of TM1088 proteins

**Fig 2:**
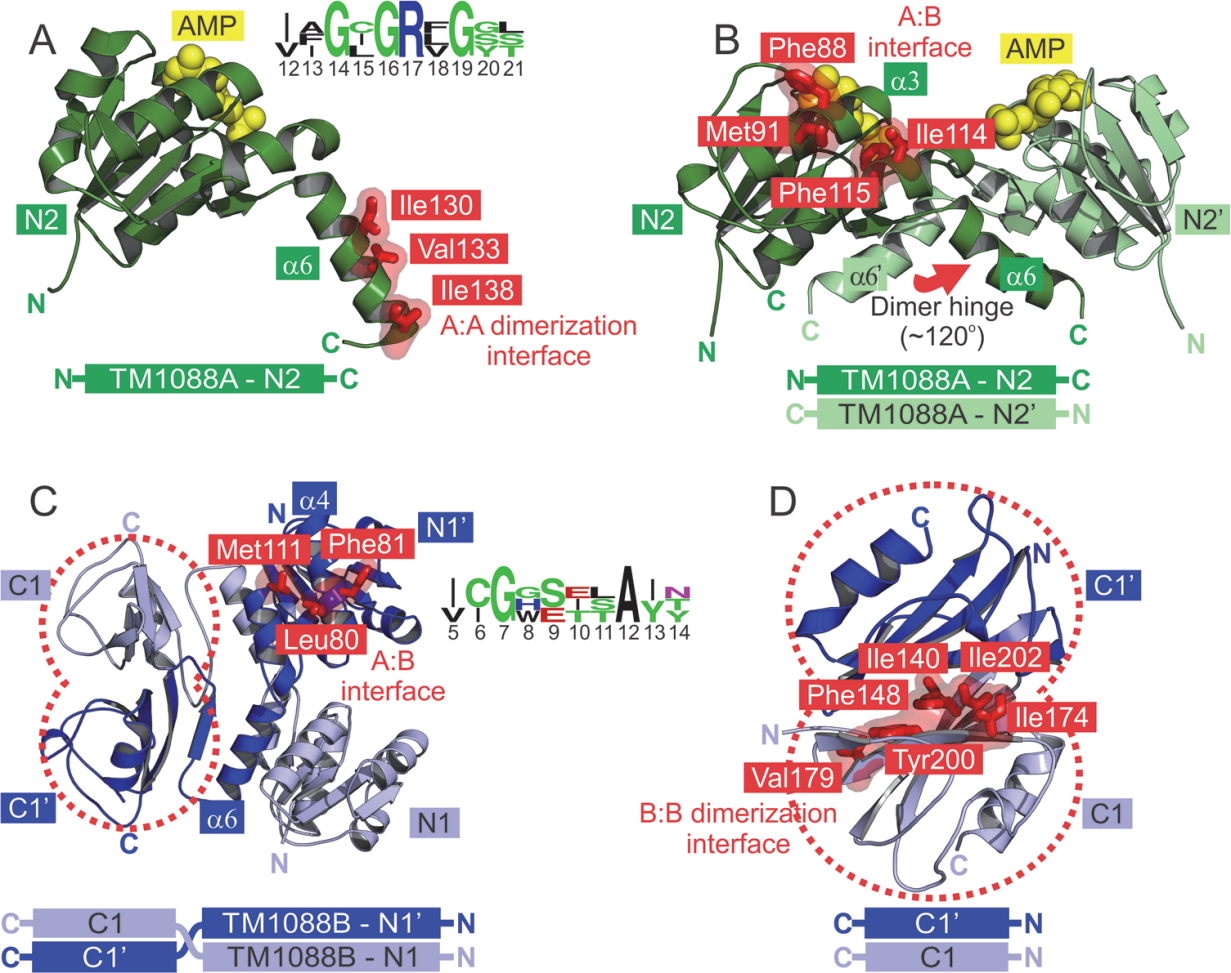
Crystal structures of TM1088 proteins. (A) Ribbon representation of the high-resolution structure of TM1088A showing some of the conserved hydrophobic residues involved in dimer formation (Ile130, Val133 and Ile138 as red sticks. Bound AMP is displayed as yellow spheres. Sequence logo of the nucleotide binding motif region (Gly14/Gly16/Gly19) from structural alignment with other nucleotide binding RCK family members is shown inset (PDB ID: 1LSS and 1LSU). Each position of the sequence is represented by a stack of residues with the height of each stack representing the conservation at that position. Glycine and polar amino acids are colored green, basic amino acids are colored blue and hydrophobic amino acids are colored black [47]. (B) Ribbon representation of the TM1088A dimer of the octameric TM1088 assembly. Conserved hydrophobic residues involved in the TM1088A to TM1088B interface are shown as red sticks (Phe88, Met91, Ile114 and Phe115). The “dimer hinge” angle is denoted by a red arrow. (C) Ribbon representation of the TM1088B dimer from the octameric TM1088 assembly. Conserved hydrophobic residues involved in the TM1088A to TM1088B interface are shown as red sticks (Leu80, Phe81 and Met111). Sequence logo of the structurally equivalent region to the AMP binding site of TM1088A is shown inset. A structural alignment of non-nucleotide binding RCK family members (PDB ID: 1ID1 and 1LNQ) was used for the alignment. (D) Ribbon representation of the TM1088B C-terminal domain. Conserved hydrophobic residues involved in the TM1088B dimer interface are shown as red sticks (Ile140, Phe148, Ile174, Val179, Tyr200 and Ile202).

The crystal structures of full-length TM1088A (Fig 2A–2B and Table 1, PDB ID: 2G1U) and the C-terminal domain of TM1088B (Fig 2C–2D and Table 1, PDB ID: 3JXO) were determined to 1.50 Å and 1.55 Å, respectively. The overall fold, dimer-interfaces and arrangement of these higher resolution structures are consistent with the corresponding components in the octameric TM1088 assembly.

TM1088A contains one molecule in the asymmetric unit (domain N2, Fig 2A) that forms a canonical N-terminal Trk domain comprising of a domain-swapped dimer in the crystal lattice (domains N2 and N2’, Fig 2B). It is important to note that each chain can be considered a single RCK unit, and for consistency with the literature and previous structural studies, two RCK units form the canonical standard assembly. This distinction is important as other K^+^ transporters achieve the same quaternary structure as TM1088 proteins using a single polypeptide chain. This same dimeric arrangement is also observed in the octameric TM1088 assembly. TM1088A adopts a Rossmann-like fold with each domain consisting of a central core of six-stranded parallel β-sheets (β1-β6) flanked by three α-helices on one side (α3, α4 and α5) and three on the other side (α6’, α1 and α2). Each domain also contains a characteristic GXGXXG nucleotide binding site motif (Fig 2A) consistent with a role in nucleotide binding.

Similarly, TM1088B also adopts a Rossmann-like fold and forms a similar dimer in both the TM1088 complex and the high-resolution structure (Fig 2C). In contrast, each chain of TM1088B consists of two sub-domains; a Trk N-terminal domain (N1 and N1’) corresponding to Val3-Gly112 and a TrkA C-terminal domain (C1 and C1’) spanning Glu143-Leu215. TM1088B does not possess a dinucleotide binding site (corresponding region shown as a sequence logo in Fig 2C). The C-terminal Trk domain (C1 and C1’) was also determined independently to high-resolution and forms a two-layer sandwich structure referred to as an alpha-beta plait (CATH classification 3.30.70, Fig 2D).

**Fig 3:**
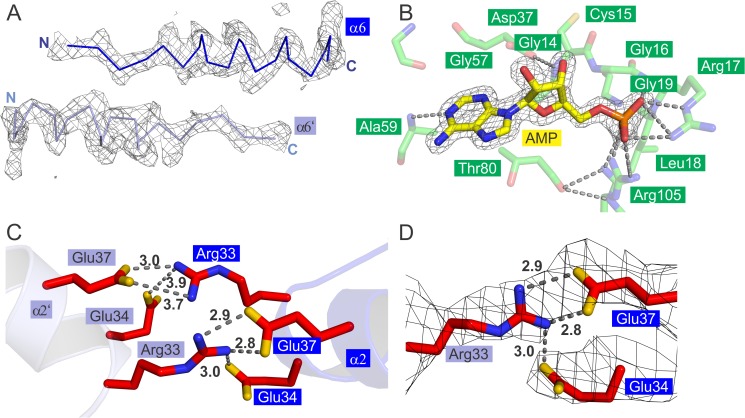
Nucleotide binding and other structural features of TM1088 proteins. (A) TM1088B inter-domain α6 helix between Thr114 and Pro133. The refined 2F_o_-F_c_ electron density map is contoured at 1σ. (B) TM1088A nucleotide binding site. AMP is shown as yellow sticks in the high-resolution TM1088A structure. Conserved TM1088A residues interacting with AMP are displayed as green sticks (oxygen atoms are red, nitrogen blue, phosphate orange and sulfur yellow). Key hydrogen bonds are shown by gray dashed lines. A sigma-A weighted omit F_o_-F_c_ electron density map of the bound AMP is contoured at 2.5σ. (C) TM1088B interfacial hydrogen-bonding pattern. Residues involved in hydrogen bonding across the central cavity of the octameric TM1088 assembly (Arg33, Glu34 and Glu37) are shown as red sticks (oxygen atoms are colored yellow and nitrogen blue). Hydrogen bonds are shown as gray dashed lines. (D) Half of the interfacial hydrogen bonding pattern is shown (for clarity) with a refined 2F_o_-F_c_ electron density map contoured at 1σ.

The N1 and C1 Trk domains of TM1088B are connected via an inter-domain linker (Ile113-Leu142) that consists predominantly of α6 of the N-terminal Trk domain (highlighted in Fig 2C) with good electron density throughout (Fig 3A). This region is important for arrangement and dimerization of the C-terminal Trk domains, and in a similar fashion to the structurally equivalent α6 of TM1088A, is responsible for the domain-swapped dimer of TM1088B. The angle between helices α6 of the dimer pairs of TM1088A and TM1088B form what is commonly referred to as the “dimer hinge” angle (highlighted by red arrow in Fig 2B). This “dimer hinge” angle is central to the gating ring mechanism in the family of RCK proteins.

The octameric TM1088 complex has three main protein:protein interaction surfaces: 1) TM1088A:TM1088A dimerization interface, 2) TM1088B:TM1088B dimerization interface and 3) TM1088A:TM1088B interface (Table 2, conserved residue involved in these interaction are highlighted as red sticks in Fig 2A–2D). The TM1088A dimer is held together by the hydrophobic C-terminal portion of α6 (Pro125-Ile138) of each monomer, which packs against portions of α1 (Arg17-Ser28) and β4 (Met75-Ala78), β5 (Asn101-Arg105) and β6 (Lys121-Ile123) of the adjacent subunit. Conserved residues central to TM1088A dimerization include Ile130, Val133 and Ile138 (red sticks in Fig 2A). The TM1088A dimer interface is predominantly hydrophobic with ~1600 Å^2^ of total buried surface area involving 39 non-bonded contacts over 22 interface residues (Table 2).

**Table 2:** Interactions in the three main protein interfaces of the octameric TM1088 assembly.

TM1088 interface	PDB Chains	Interface residues of first component	Interface residues of second component	Interface area of first component (Å^2^)	Interface area of second component(Å^2^)	H-bonds in interface	Non-bonded contacts
**A:A**	**BE** *	**11**	**11**	**813**	**823**	**-**	**39**
**A:A**	**AF** *	**11**	**10**	**770**	**797**	**-**	**34**
**B:B**	**CD** *	**28**	**29**	**2099**	**2070**	**10**	**129**
**B:B**	**GH** *	**24**	**24**	**1712**	**1756**	**7**	**100**
B:B	DH	3	2	104	132	2	23
B:B	CG	1	1	56	60	-	4
**A:B**	**BD** *	**15**	**13**	**766**	**774**	**4**	**91**
**A:B**	**AC** *	**14**	**13**	**761**	**749**	**3**	**64**
**A:B**	**EG** *	**13**	**13**	**721**	**722**	**3**	**76**
**A:B**	**FH** *	**11**	**11**	**705**	**701**	**2**	**52**
A:B	FG	2	3	118	111	1	14
A:B	EH	2	3	112	103	-	18
A:B	AD	1	3	109	100	-	11
A:B	BC	1	3	109	100	-	13

*bold denotes dimer pairs within the octameric TM1088 assembly. Data were calculated using PDBsum for entry 3l4b [48].

Similar regions of the TM1088B N-terminal domain are also involved in dimerization, specifically the structurally equivalent α6 helix that forms the inter-domain linker. However, further interactions are also facilitated by the additional C-terminal Trk domain. These interactions predominantly involve conserved hydrophobic residues Ile140, Phe148, Ile174, Val179, Tyr200 and Ile202 that are all situated on the central β-sheet of the Rossmann-like fold (red sticks in Fig 2D). As a consequence of this extra dimerization domain, the surface buried in the TM1088B dimerization interface is significantly larger than that of the TM1088A dimer with over 4000 Å^2^ of total buried surface area involving 129 non-bonded contacts over 57 residues. Additionally, the TM1088B dimerization interface involves 10 hydrogen bonds that are not observed in the TM1088A dimerization interface (Table 2) and are predominantly located at the interface between the N-terminal Trk domain (Arg17 and Ser18) and the inter-domain α6 helix (Asp101, Thr124 and Glu128).

The third major protein interface is between adjacent TM1088A and TM1088B dimer pairs. This hydrophobic surface is composed of TM1088A residues on α3 (Asp83-Phe 97) and α4 (Ile112-Asn118) and TM1088B residues on α4 (Ile82-Asp76) and α6 (Ile107-Met111). Conserved residues buried in this interface include TM1088A Phe88, Met91, Ile114 and Phe115 (highlighted by red sticks in Fig 2B) and TM1088B Leu80, Phe81 and Met111 (highlighted by red sticks in Fig 2C). This is the smallest of the three protein interfaces in the octameric TM1088 assembly involving ~1500 Å^2^ of total buried surface area and 91 non-bonded interactions and a total of 28 residues (Table 2). This interface is structurally equivalent to the “helix crossover” motif, which has been previously shown to be central to the gating ring mechanism in the RCK family of proteins.

Overall, the protein:protein interfaces of the TM1088 assembly are extensive (just under 18,000 Å^2^ of total buried surface area), specific (32 hydrogen bonds) and structurally homologous to interfaces observed in other K^+^ gating ring assemblies, further indicating the partnering of the components is highly specific and correctly assembled (Table 2).

### Nucleotide binding and other structural features of TM1088 proteins

AMP was co-purified with TM1088A and appears in both the high-resolution TM1088A structure and the low-resolution structure of the octameric TM1088 assembly (AMP shown as yellow spheres or sticks in Figs 1C, 2A–2B and 3B).

AMP was not included in the crystallization reagents and was presumably scavenged from the *E*. *coli* cells during expression. NADH was included in the crystallization reagents as a result of database annotations that suggest NADH as a potential ligand. Previous differential scanning calorimetry (DSC) and Thermofluor experiments conducted in our lab showed that adenosine-containing nucleotides are capable of binding to TM1088A [9]. Specifically, AMP stabilized the TM1088A melting temperature by +5.8^°^C (DSC) and +8^°^C (Thermofluor) and NADH by +2.3^°^C (DSC) and +4^°^C (Thermofluor). AMP showed the highest degree of stabilization for all adenosine nucleotides tested and no stabilization of TM1088B was observed as expected [9]. DSC and Thermofluor analysis could not be performed on the TM1088 complex, as a result of a melt profile in excess of 130^°^C, which is outside the range of the DSC and Thermofluor equipment.

Residues involved in nucleotide interaction include the highly conserved loop region between α1 and β1 that contain the nucleotide binding motif (Gly14-X-Gly16-X-X-Gly19) and additional residues, including Asp37, Gly57, Ala59, Thr80 and Arg105 (Fig 3B). Key conserved residues involved in hydrogen bonding with the AMP include Ala59, Arg17 and Arg105, which function in anchoring the AMP at opposite ends via the adenine and phosphate moieties of the AMP (Fig 3B). The AMP is located in a similar position and orientation to the adenosine-containing nucleotides bound in other KtrAB and TrkA structures [20,21].

One unique and distinguishing feature of the TM1088 assembly is the pattern of the hydrogen-bonding network observed across the central cavity between opposing TM1088B dimers (highlighted in red in Fig 1C) as a pseudo-symmetrical arrangement formed by Arg33 of helix α2 with Glu37 and Glu34 of the opposing α2’ (Fig 3C). This interaction displays good electron density and, therefore, must be highly ordered (Fig 3D).

### TM1088 proteins are structurally homologous to each other and to other RCK domains

**Fig 4:**
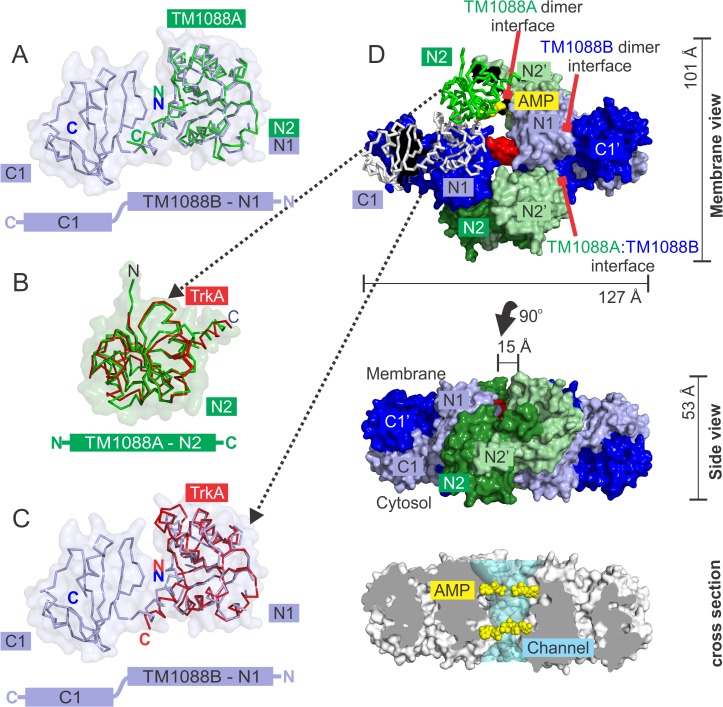
TM1088 proteins are structurally homologous to each other and to other RCK domains. (A) Superposition of the N-terminal Trk domain of TM1088A (N2, green) onto the N-terminal Trk domain of TM1088B (N1, blue). (B) Superposition of the N-terminal Trk domain of TM1088A (N2, green) onto the N-terminal TrkA domain of *V*. *parahaemolyticus* (red, PDB ID: 4J9U). (C) Superposition of the N-terminal Trk domain of TM1088B (N1, blue) onto the N-terminal TrkA domain of *V*. *parahaemolyticus* (red, PDB ID: 4J9U). (D) Solvent accessible surface of the octomeric TM1088 assembly with the surface stripped away from the N1, N2 and C1 domains to show the underlying Cα trace used in the alignments in panels A-C. AMP is shown as yellow spheres. The solvent accessible channel traversing the assembly is shown in aqua. Residues involved in hydrogen bond formation across the central channel are highlighted by a red surface (Arg33, Glu34 and Glu37). The three types of protein interface in the assembly are highlighted by red arrows.

Although TM1088A and TM1088B share only 23% sequence identity, they are structurally homologous to each other (RMSD of 1.2 Å over 122 aligned Cα atoms, Fig 4A). TM1088A is shorter and consists only of an N-terminal TrkA domain and linker region. Given the somewhat atypical nature of the two-subunit TM1088 assembly, we conducted extensive structural superpositions and DALI searches to identify the closest structural homologs of TM1088 proteins [49]. TM1088 proteins are structurally similar to all previously determined RCK structures with the best structural alignment occurring with the N-terminal Trk domain of TM1088A (N2) and TrkA of *V*. *parahaemolyticus* (Fig 4B, PDB ID: 4J9V, RMSD of 1.2 Å over 71 Cα atoms)[21]. The same TrkA domain also aligns well with the N-terminal Trk domain (N1) of TM1088B (Fig 4C, PDB ID: 4J9V, RMSD of 1.0 Å over 72 Cα atoms). Other notable structural alignments of TM1088A include those with RCK domains of TrkA from *M*. *jannaschii* (PDB ID: 1LSS, RMSD of 1.1 Å over 99 Cα atoms)[19], KtrA of *B*. *subtilis* (PDB ID: 1LSU, RMSD of 3.8 Å over 81 aligned residues)[19], MthK of *M*. *thermautotrophicus* (PDB ID: 1LNQ, RMSD of 1.6 Å over 92 aligned atoms)[16] and BK of *Danio rerio* (PDB ID: 3U6N, RMSD of 1.90 Å over 93 Cα atoms)[21]. It is important to note that full-length, non-flexible superpositions of TM1088B (N1 and C1 domains together) do not result in good alignments with other RCK proteins as a consequence of different relative positions of the N- and C-terminal RCK domains (N1 and C1) and changes in the inter-domain linker regions and domain-swapping.

In addition, the quaternary structure, domain arrangement, overall shape and size is similar to that of other RCK gating ring assemblies, such as TrkA (PDB ID: 4J9U)[21], MthK (PDB ID: 2FY8)[16,18], KtrA (PDB ID: 2HMS and 4J7C)[20,23], GsuK (PDB ID: 4GX5)[25] and BK (3U6N and 3NAF)[22,24]. TM1088 homodimers are assembled in a pseudo-tetrameric arrangement to generate an octameric assembly with approximate dimensions of 127 Å x 101 Å x 53 Å (Fig 4D). Moreover, similar homodimeric arrangements are observed in the non-complexed high-resolution crystal structures of TM1088A (PDB ID: 2G1U) and TM1088B (PDB ID: 3JXO) further supporting the correct assembly of these dimers into an octameric arrangement. The N-terminal Trk domains of TM1088B (N1) and TM1088A (N2) form the two sides of the ring, while the C-terminal Trk domains of TM1088B (C1) are peripheral and lie outside the central ring (Fig 4D).

The TM1088 assembly forms a large central channel with a total solvent accessible surface of ~9700 Å^2^ (calculated by CASTp and MOLE [50,51], channel shown as aqua surface in Fig 4D). The channel is ~50 Å in length and is formed by a roughly equal contribution of residues from all 8 chains and is readily accessible to solvent on both ends. The four AMP molecules reside on the inside of the channel and are solvent accessible. The AMP molecules are bound to the TM1088A N-terminal TrkA domain (N2) at positions flanking the dimer interface. The AMP molecules line the central channel in a similar position to nucleotides observed in both KtrA and TrkA gating ring assemblies (AMP shown as yellow spheres in Fig 4D). The central channel of the TM1088 complex is ~15 Å at its narrowest, which is comparable to the ~15 Å diameter at the center of the MthK channel (PDB ID: 2FY8)[18].

### TM1088 assembly has an RCK domain arrangement homologous to other gating ring assemblies

**Fig 5:**
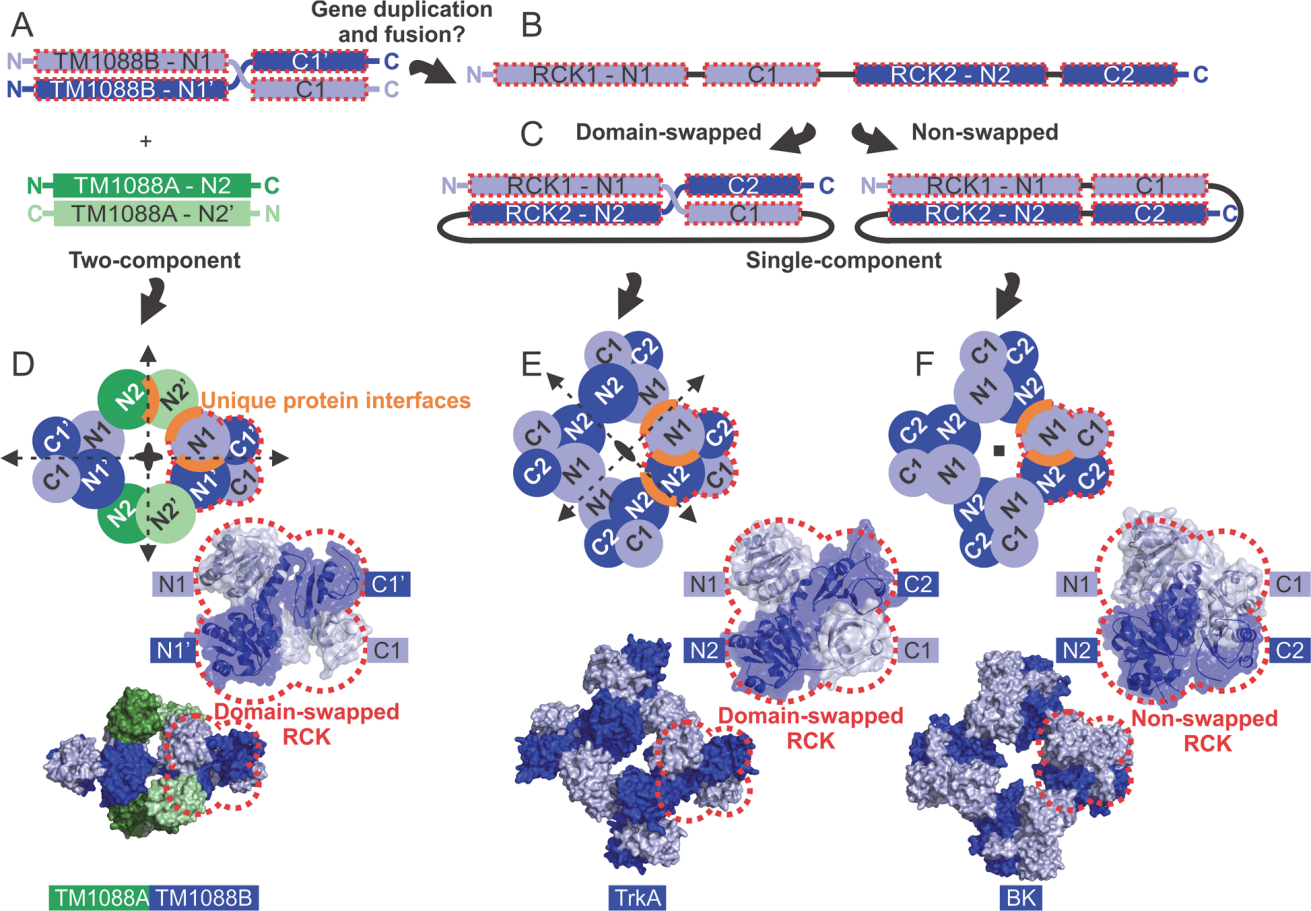
TM1088 assembly has an RCK domain arrangement homologous to other gating ring assemblies. (A) Schematic arrangement of the N-terminal (N1 and N2) and C-terminal (C1) RCK domains of TM1088A (green) and TM1088B (blue). (B) Possible gene duplication and fusion event resulting in a more classical single-chain arrangement as observed in other K^+^ gating ring assemblies. (C) Two-pathways of protein assembly resulting in a domain-swapped RCK or a non-swapped RCK. (D) Top: Schematic of the domain organization of the TM1088 assembly, showing the two-fold symmetry elements (black arrows) and the unique protein interfaces (orange curves). Middle: The repeating TM1088B RCK unit highlighting the domain-swapped organization. Bottom: molecular surface of the TM1088 assembly showing the corresponding domains. (E) Top: Schematic of the domain organization of the TrkA assembly (PDB ID: 4J9U), showing the two-fold symmetry elements (black arrows) and the unique protein interfaces (orange curves). Middle: The repeating TrkA RCK unit highlighting the domain-swapped organization. Bottom: molecular surface of the TrkA assembly showing the corresponding domains. (F) Top: Schematic of the domain organization of the BK assembly (PDB ID: 3U6N), showing the four-fold symmetry element (black square) and the unique protein interfaces (orange curves). Middle: The repeating BK RCK unit highlighting the non-swapped domain organization. Bottom: molecular surface of the BK assembly showing the corresponding domains. Panels E-F adapted from [21].

As discussed above, the best structural alignments for both TM1088A and TM1088B are achieved with the N-terminal domain of TrkA from *V*. *parahaemolyticus* (Fig 4B–4C). Differences in the overall quaternary structure of TM1088 and TrkA are a result of the atypical two-subunit nature of the TM1088 assembly and the use of a domain-swapped organization. We have made an attempt to place the two-subunit TM1088 assembly into a more standard framework in line with previously studied single-subunit K^+^ transporter gating ring assemblies as detailed in Fig 5. It is important to note that all structures of RCK domains determined to date involve similar domain swapping; in the case of TM1088A secondary structure elements are swapped between monomers (i.e. helix 6), whereas in TM1088B, entire tertiary structure elements are swapped between monomers (i.e. N-terminal and C-terminal domains).

We propose that a simple gene duplication and fusion event (Fig 5A–5B), followed by different patterns of domain organization (Fig 5C), may be sufficient to account for the differences between the two-subunit TM1088 assembly and other single-subunit K^+^ gating ring assemblies (Fig 5D–5F). Duplication and fusion of the two-chains of TM1088B into a consolidated polypeptide chain consisting of two-tandem RCK repeats would be one possible route (Fig 5B, RCK1 and RCK2 shown, each with an N-terminal and C-terminal domain). Obviously, other combinations of duplication and fusions utilizing both TM1088A and TM1088B could also be envisaged.

Previous structural studies have all shown that gating ring structures are assembled from a total of eight central RCK domains, with the central channel formed by an N-terminal domain of the RCK unit (Fig 5D–5F, N-terminal domains denoted as N1 and N2)[16,18,20–25]. However, in contrast, the N1 and N2 domains of TM1088 are not housed on a single chain, but instead are spread across the two homodimers of TM1088A and TM1088B, respectively (N1, N1’ and N2, N2’, respectively. Fig 5D). This arrangement forms a symmetric repeating RCK unit around the central channel. Comparison of this repeating RCK unit with that of the gating ring assemblies of TrkA and BK reveals that two different patterns of domain organization are used within the RCK unit (red dotted lobes in Fig 5D–5F). For TM1088, the N1 domain is on the opposite side to the C1 domain and, as such, forms a domain-swapped RCK (Fig 5D). This is comparable to the more conventional single-subunit TrkA system, in which the analogous N1 and C1 domains are also on opposite sides of the protein (Fig 5E). However, other K^+^ gating ring complexes, such as BK (and Mthk), do not adopt a domain-swapped organization and the N1 and C1 domains lie on the same side of the protein (Fig 5F).

The organization of the RCK domains results in some interesting differences in symmetry. The TM1088 assembly has *D*_*2*_ point symmetry involving four, perpendicular and intersecting, two-fold rotational axes of symmetry (Fig 5D) and is similar to the *D*_*2*_ point symmetry of TrkA, which involves three, perpendicular and intersecting, two-fold rotational axes of symmetry (Fig 5E). However, the BK channel (and MthK) has *C*_*4*_ symmetry as a result of the lack of a domain-swapped organization (Fig 5F). It is important to note that similar *C*_*4*_ symmetry occurs in the non-domain swapped potassium channel GsuK [25] and, therefore, the model presented in Fig 5 may only pertain to transporters of specific families. However, to determine if any global rules exist that relate symmetry and the RCK domain architecture, further gating ring structures will be required. As a result of the symmetry, the TM1088 assembly has three unique protein:protein interfaces (N1:N1’, N2:N2’ and N1:N2’ highlighted by orange curves in Fig 5D), in common with TrkA, which also has three unique protein:protein interfaces (N1:N1, N1:N2 and N2:N2 highlighted by orange curves in Fig 5E). However, in contrast, the BK channel (and Mthk) only has two unique protein:protein interfaces (N1:N2 and N2:N1 highlighted by orange curves in Fig 4F)[21].

### Potential role of TM1088 interfaces in the gating ring assembly

**Fig 6:**
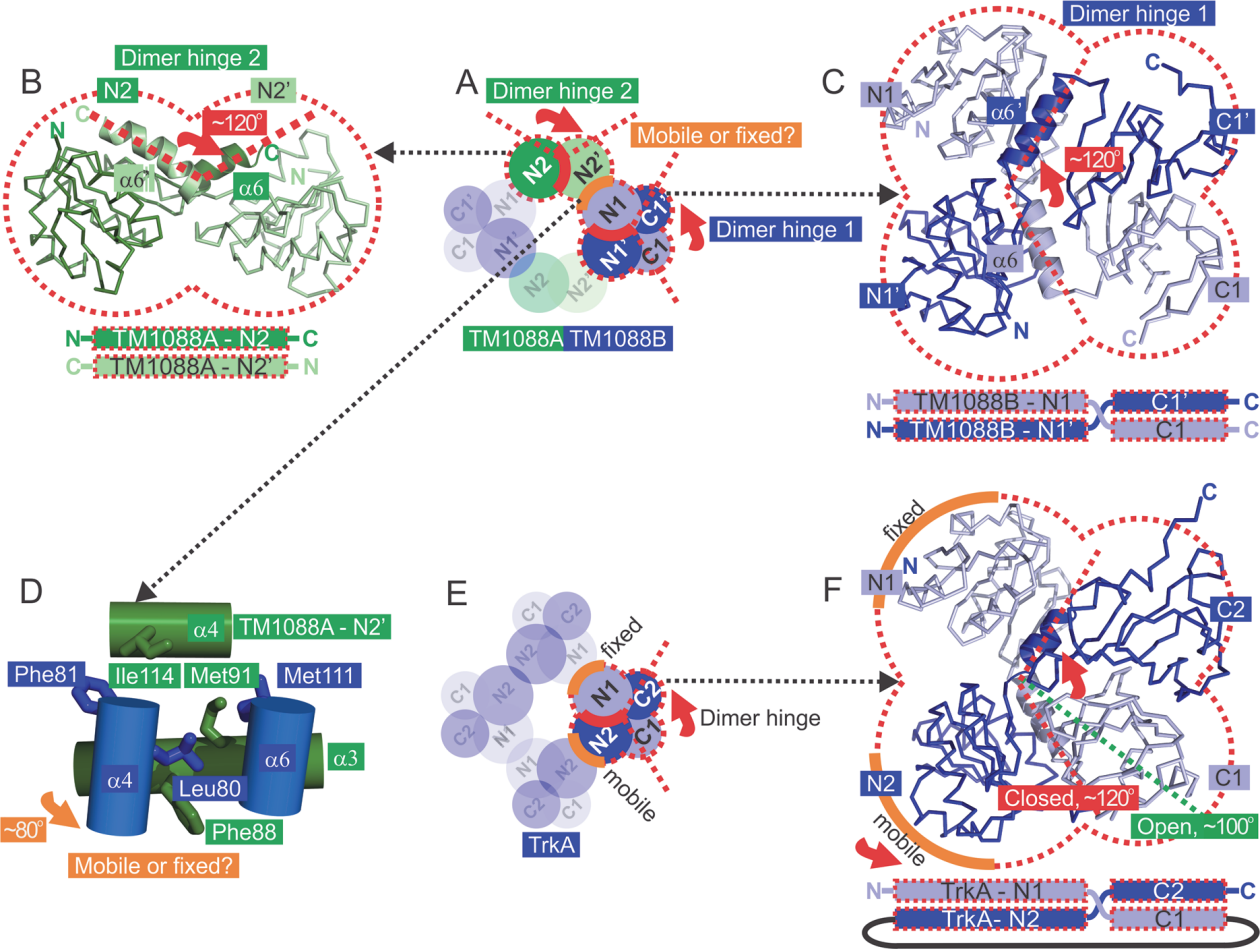
Potential role of TM1088 interfaces in the gating ring assembly. (A) The two unique dimers of the TM1088 assembly are highlighted (red dashed lobes) showing the three unique protein:protein interfaces (orange and red curves). The “dimer hinge 1” between TM1008B dimer partners (red arrow), “dimer hinge 2” between TM1088A dimer partners (red arrow) and the “mobile or fixed” interface between adjacent dimer pairs (orange curve) are shown. (B) “Dimer hinge 2” between the N-terminal Trk domains N2 and N2’ of TM1088A. (C) “Dimer hinge 1” between N-terminal Trk domains N1 and C1, and N1’ and C1’ of TM1088B. (D) “Mobile or fixed” interface between adjacent TM1088A and TM1088B dimer pairs. TM1088A helices are shown in green (α3: Asp83-Phe97 and α4: Ile112-Asn118) and TM1088B helices in blue (α4: Ile82-Asp76 and α6: Ile107-Met111). Conserved hydrophobic residues are shown as green sticks (TM1088A: Phe88, Met91 and Ile114) and blue sticks (TM1088B: Leu80, Phe81, Met111). The “helix crossover” angle between α3 of TM1088A and α4 of TM1088B is denoted by an orange arrow. (E) The single unique dimer of the TrkA assembly (PDB ID: 4J9U) is highlighted showing the three unique protein interfaces (orange and red curves). The “dimer hinge” angle between dimer partners for the closed conformation (red dashed line, ADP-bound TrkA, PDB ID: 4J9U) and the open conformation (green dashed line, ATPγS-bound TrkA, PDB ID: 4J9V) are shown.

Surprisingly, the atypical two-subunit structure of the TM1088 assembly results in three unique protein:protein interfaces that are all structurally homologous to interfaces known to be central to gating ring assembly in the RCK family of K^+^ transport proteins (Fig 6A). These interfaces are classically characterized as being either a) an intra-chain flexible “dimer hinge” between RCK domains or b) a “mobile” or “fixed” inter-chain interface between neighboring RCK domains. Interestingly, as a result of its two-subunit nature, the TM1088 assembly has an additional “dimer hinge” not observed in other gating ring assemblies (Fig 6A). The N1:N1’ interface of the TM1088B dimer forms “dimer hinge 1” that is analogous to the more classical dimer hinge between the N- and C-terminal domains as observed in TrkA, BK and MthK assemblies. The N2:N2’ interface of the TM1088A dimer forms “dimer hinge 2” (Fig 6A). The third interface between adjacent TM1088A and TM1088B dimer pairs (N1:N2’) is structurally analogous to the “mobile” or “fixed” interfaces of other K^+^ gating ring assemblies (Fig 6A). The “dimer hinge” angles of TM1088A and TM1088B are ~120° (Fig 6B and 6C, respectively). The “mobile or fixed” interface of TM1088, which can be measured as the “helix-crossover” angle between TM1088B and TM1088A dimers is measured as ~80° (Fig 6D).

It is important to note that, as a result of the lower *D*_*2*_ symmetry, the TrkA (and MthK) assemblies consist of an alternating pattern of “fixed” and “mobile” interfaces around the central channel (Fig 6E). In contrast, the BK (and KtrAB) assemblies have higher *C*_*4*_ symmetry and, therefore, possess only a single type of “mobile” interface.

## Conclusions

The protein products of locus *TM1088* assemble to form an octameric cytosolic regulatory component that likely represents the Trk homolog in *T*. *maritima*. The TM1088 assembly is the first K^+^ transporter structure assembled from two different, but structurally homologous proteins. The TM1088 assembly consists of the same folds, domains and assembly motifs as other K^+^ transporter assemblies, such as TrkA, GsuK, KtrAB, MthK and BK [16,18,20–22,24,25]. Given the atypical genetics and dimer composition of the TM1088 assembly, it is somewhat remarkable that it contains the same motifs that define the regulation paradigm of this family of RCK domain containing channels.

The differences between the two-subunit TM1088 assembly and other more classical single-subunit K^+^ gating ring assemblies can be understood by a simple gene duplication and fusion event (Fig 5), as proposed for the evolution of *E*. *coli* TrkA and other Trk/KtrAB proteins [14,26]. Furthermore, additional regulatory units have also been identified in the Trk/Ktr transporters of *E*. *coli* (TrkA and TrkE), *Synechocystis sp*. *PCC6803* (KtrA and KtrE) and *B*. *subtilis* (KtrA and KtrC)[6]. Additionally, *T*. *maritima* is one of the oldest known bacteria with a very deep lineage and a propensity for lateral gene transfer [52,53]. Sequences of the 16S rRNA gene of members of the *Thermotogales* phylum reveal similarities to the last common ancestor and also suggest that the phylum is the closest bacterial relative to archaea bacteria and eukarya [54].

Homologs of the two-subunit Trk system can also be found in several other bacterial genomes including *Actinobacteria* and *Dehalococoides*, illustrating their central role in bacterial physiology [9,55]. Most significantly, a two-subunit TrkA homolog has also been identified in *Mycobacterium tuberculosis* [9] and represents a novel target for structure-based drug design and the development of new anti-mycobacterial agents against multidrug-resistant strains. Members of the Trk/Ktr/HKT family are only found in non-animal cells and have no close homologs in animals and, therefore, represent highly selective targets for drug action [6]. Several anti-mycobacterials function by targeting K^+^ uptake, including clofazimine and 7-methyljuglone, although little is known about their mechanisms of action [56,57].

A key structural feature of the TM1088 assembly is the presence of interfacial hydrogen bonds across the central channel. It is possible that these hydrogen bonds are actually salt bridges, as observed in the gating function of the bacterial ion-channel OmpA [58]. The gate of the OmpA channel is formed by a central salt bridge (Glu52-Arg138), which can flip-flop between two discrete states that represent closed and open forms [59]. Furthermore, Arg33 of TM1088B is structurally equivalent to Glu146 of the MthK assembly, which is also involved in the formation of interfacial contacts between neighboring RCK subunits upon gating ring assembly [18]. It is also possible that these interactions function in stabilizing the complex in this thermophilic organism. The contribution of such salt-bridge interactions to protein stability has been debated in previous studies and it has been suggested that thermophilic analogues of mesophilic proteins possess an increased number of salt bridges [60–62]. However, mutational studies suggest the contribution to protein stability is relatively small, although their stabilizing effects may be more apparent at higher temperatures [63]. Cross-pore inter-subunit hydrogen bonds of this type have not been observed in previously reported KtrA or TrkA structures, although salt bridges are formed between the cytosolic TrkA and the membrane component TrkH [20,21].

The central dogma of gating ring assembly and regulation of ion-flow by the K^+^ family of transporters is the expansion or contraction of the central ring of N-terminal RCK domains. [16,18,20–25]. This movement is largely effected via conformational changes in the “dimer hinge” region between the N- and C-terminal RCK domains. To accommodate the movement of these domains, the neighboring N-terminal RCK domains must also move, as demonstrated for the BK and Mthk gating ring assemblies, in which the protein interfaces on either side of the “dimer-hinge” are all the same and “mobile” in nature [16,18,22,24]. The ring then accommodates changes in the position of the N-terminal RCK domain using a uniform, four-fold symmetric dilation of the gating ring. However, in contrast, as a result of the domain-swapped nature of their dimer pairs, which in turn leads to a lower symmetry of domains around the central ring TM1088, TrkA and KtrAB gating ring assemblies contain two-distinct protein interfaces on either side of the “dimer hinge” [20,21,23]. One of these interfaces forms a “mobile” interface (analogous to the single “mobile” interface of BK and MthK) and the second distinct interface forms a “fixed” interface that does not move to accommodate changes in the “dimer-hinge” (Fig 6E).

It is likely that regulation of ion-flow by the TM1088 assembly could occur using similar “dimer hinge” and “mobile” interfaces, although the presence of a “fixed” interface has yet to be determined. It is important to note that at least one interface must be “mobile” to accommodate expansion or contraction of the central ring. However, given the high-level of structural homology with the TrkA gating ring assembly, and the similar *D*_*2*_ symmetry, it is likely that the TM1088 assembly will function by using two distinct “mobile” and “fixed” interfaces. The “dimer hinge” angles of the TM1088 assembly are ~120°, which is also in agreement with the angles observed in the ADP-bound, and presumably closed form, of the TrkA/TrkH gating ring complex from *V*. *parahaemolyticus*. These angles compare with the ATPγS-bound, and presumably open conformation, of TrkA/TrkH which has a “dimer-hinge” angle of ~100° (Fig 6E)[21].

The similarities between the ADP-bound TrkA/TrkH structure and the AMP-bound TM1088 assembly suggests that our structure most closely resembles a closed state of the channel. To confirm this hypothesis, further biochemical studies will be required. Additionally, to confirm the exact nature of each interface, and the mechanism of regulation, additional structures of the TM1088 assembly captured in various conformational states will also be needed. Perhaps more importantly, high-order assemblies of TM1088 in association with its membrane-spanning TrkH component, TM1089, will also be required to address the mechanism of coupling between the cytosolic gating ring component and that of the membrane-spanning channel.

Finally, two-subunit Trk proteins are predicted to be fairly commonplace in the bacterial realm [6,9]. The structure of the TM1088 assembly provides the first structural insights into what may be an evolutionary ancestor of more modern single-subunit gating ring assemblies.
